# Dyslipidemia and serum cystatin C levels as biomarker of diabetic nephropathy in patients with type 2 diabetes mellitus

**DOI:** 10.3389/fendo.2023.1124367

**Published:** 2023-04-04

**Authors:** Tadesse Asmamaw Dejenie, Endeshaw Chekol Abebe, Misganaw Asmamaw Mengstie, Mohammed Abdu Seid, Natnael Atnafu Gebeyehu, Getachew Asmare Adella, Gizchew Ambaw Kassie, Amanuel Yosef Gebrekidan, Molalegn Mesele Gesese, Kirubel Dagnaw Tegegne, Denekew Tenaw Anley, Sefineh Fenta Feleke, Melkamu Aderajew Zemene, Anteneh Mengist Dessie, Natnael Moges, Yenealem Solomon Kebede, Berihun Bantie, Dagnew Getnet Adugna

**Affiliations:** ^1^ Department of Medical Biochemistry, College of Medicine and Health Sciences, University of Gondar, Gondar, Ethiopia; ^2^ Department of Biochemistry, College of Health Science, Debre Tabor University, Debre Tabor, Ethiopia; ^3^ Department of Physiology, College of Health Science, Debre Tabor University, Debre Tabor, Ethiopia; ^4^ Department of Midwifery, College of Medicine and Health Science, Wolaita Sodo University, Sodo, Ethiopia; ^5^ Department of Reproductive Health and Nutrition, School of Public Health, Woliata Sodo University, Sodo, Ethiopia; ^6^ Department of Epidemiology and Biostatistics, School of Public Health, Woliata Sodo University, Sodo, Ethiopia; ^7^ Department of Public Health, School of Public Health, College of Health Sciences and Medicine, Wolaita Sodo University, Sodo, Ethiopia; ^8^ Department of Nursing, College of Medicine and Health Science, Wollo University, Dessie, Ethiopia; ^9^ Department of Public Health, College of Health Sciences, Debre Tabor University, Debre Tabor, Ethiopia; ^10^ Department of Public Health, College of Health Sciences, Woldia University, Woldia, Ethiopia; ^11^ Department of Pediatrics and Child Health Nursing, College of Health Sciences, Debre Tabor University, Debre Tabor, Ethiopia; ^12^ Department of Medical Laboratory Science, College of Health Sciences, Debre Tabor University, Debre Tabor, Ethiopia; ^13^ Department of Comprehensive Nursing, College of Health Sciences, Debre Tabor University, Debre Tabor, Ethiopia; ^14^ Department of Anatomy, School of Medicine, College of Medicine and Health Science, University of Gondar, Gondar, Ethiopia

**Keywords:** diabetes, diabetic nephropathy, serum cystatin C, serum creatinine, dyslipidemia

## Abstract

**Background:**

Diabetic nephropathy is a leading cause of end-stage renal disease. The diagnostic markers of nephropathy, including the presence of albuminuria and/or a reduced estimated glomerular filtration rate, are not clinically ideal, and most of them are raised after a significant reduction in renal function. Therefore, it is crucial to seek more sensitive and non-invasive biomarkers for the diagnosis of diabetic nephropathy.

**Objective of the study:**

This study aimed to investigate the serum cystatin C levels and dyslipidemia for the detection of diabetic nephropathy in patients with type 2 diabetes mellitus.

**Methodology:**

A hospital-based comparative cross-sectional study was conducted from December 2021 to August 2022 in Tikur, Anbessa specialized teaching hospital with a sample size of 140 patients with type2 diabetes mellitus. Socio-demographic data was collected using a structured questionnaire, and 5 mL of blood was collected from each participant following overnight fasting for biochemical analyses.

**Results:**

In type 2 diabetes patients with nephropathy, we found significant lipoprotein abnormalities and an increase in serum cystatin C (P < 0.001) compared to those without nephropathy. Serum cystatin C, systolic blood pressure, fasting blood glucose, total cholesterol, triglyceride, low density lipoprotein, very low-density lipoprotein, high density lipoprotein, and duration of diabetes were identified as being significantly associated with diabetic nephropathy (P < 0.05) in multivariable logistic regression analysis. The mean values of total cholesterol levels, triglyceride levels, and high-density lipoprotein cholesterol levels were also found to be significantly higher (P < 0.05) in females as compared to male type-2 diabetic patients. The fasting blood glucose levels and lipid profiles of the participants were found to be significantly associated with serum cystatin C levels.

**Conclusion:**

The present study found significant serum cystatin C and lipoprotein abnormalities in T2DM patients with diabetic nephropathy when compared with those without diabetic nephropathy, and these lipoprotein abnormalities were significantly associated with serum cystatin C levels.

## Background

Diabetes mellitus (DM) is a common metabolic disorder resulting from insufficient insulin secretion or insulin action that leads to elevated blood glucose levels (1). The metabolic disorders associated with DM cause secondary pathophysiological changes in multiple organ systems caused by acute and chronic complications (2). Diabetic nephropathy is one of the major chronic microvascular complications of diabetic and a leading cause of end-stage renal disease (ESRD) (3), and it is predominantly due to type 2 diabetes mellitus (T2DM) (4). Hyperglycemia is the main driving force behind the development of chronic complications of diabetic, including DKD (5). There are hypotheses that have been widely supported by scholars about how hyperglycemia causes diabetic complications (including DN), which are the polyol pathway, the hexosamine pathway (HBP), the production of AGEs, and PKC. It stimulates the resident and non-resident renal cells to produce humoral mediators and cytokines that can lead to functional and phenotypic changes in renal cells and tissues, interference with cell growth, interacting proteins, advanced glycation end products (AGEs), etc., ultimately resulting in glomerular and tubular damage and the onset of kidney disease. Therefore, poor blood glucose control is a particularly important risk factor for the development of DN.

T2DM Patients with diabetic nephropathy are often associated with abnormal lipid metabolism and lipid deposition (6). This altered lipid profile is termed “diabetic dyslipidemia,” which includes high plasma levels of total cholesterol, very low-density lipoprotein (VLDL), low-density lipoprotein (LDL), and triglycerides (TG) and a lower concentration of high-density lipoprotein (HDL). Several studies suggest that diabetic dyslipidemia plays a major role in the pathogenetic development of diabetic nephropathy in T2DM patients (7, 8). However, this has not been well studied in most of the sub-Saharan African countries, including Ethiopia.

The diagnostic markers of nephropathy, including the presence of albuminuria and/or a reduced estimated glomerular filtration rate (eGFR), are not clinically ideal, and most of them are raised after a significant reduction in renal function (9, 10). Kidney biopsy is the gold standard for differentiating diabetic nephropathy from non-diabetic nephropathy (11), but it is invasive and not suitable for routine clinical practice. Therefore, it is crucial to investigate the pathogenesis and seek more sensitive and non-invasive biomarkers for the diagnosis of diabetic nephropathy.

Human cystatin C is a member of the cystatin superfamily (inhibitors of cysteine proteinases) and a nonglycosylated basic protein with a low molecular mass (13 kD) that is freely filtered by the glomerulus and produced by all nucleated cells (12, 13). Studies have reported that unlike serum creatinine, serum cystatin C is not influenced by age, sex, muscle mass, or dietary intake (14, 15). It is currently under investigation as a promising marker and a replacement for serum creatinine and other renal markers for the early detection of renal impairment (16). Recent studies showed that cystatin C is a more sensitive serum marker for renal function assessment compared to serum creatinine (15, 17). Increased cystatin C and disorders of lipid metabolism are associated with diabetic nephropathy in T2DM patients (18). Therefore, this study aimed to explore the clinical significance of serum cystatin C and lipid profile abnormalities for the differential diagnosis of diabetic nephropathy and non-diabetic nephropathy in patients with T2DM.

## Methods and materials

### Study design, settings, and participants

A hospital-based comparative cross-sectional study was conducted from December 2021 to August 2022 in Tikur, Anbessa specialized teaching hospital, Addis Ababa, Ethiopia, with the aim of evaluating the serum cystatin C and lipid profiles as markers of diabetic nephropathy in known type 2 diabetic patients who were on regular follow-up at the diabetic clinic of Tikur, Anbessa specialized hospital. A total of 140 eligible T2DM patients who had follow-ups at the diabetic center of Tikur Anbesa specialized hospital during the study period were recruited for this study. We classified the T2DM participants into those with and without nephropathy. The first group included a total of 83 T2DM patients without nephropathy, and the second group included 57 T2DM patients with nephropathy diagnosed by physicians. We excluded patients who had any of the other complications of diabetic, medical or surgical problems, were pregnant women, or had taken lipid-lowering drugs from the study.

A structured questionnaire was used to collect socio-demographic data, including age, sex, address, history, and duration of diabetes. Anthropometric measurements such as weight, height, body mass index (BMI), and blood pressure (BP) were determined using appropriate standardized devices. The height and weight of the participants were measured following standard protocols. Then, BMI was calculated *via* weight in kilograms divided by height in meters squared (BMI = Kg/m^2^). Furthermore, blood pressure was measured using a digital automatic BP monitor apparatus for three consecutive times (five minutes apart), and the average values for both diastolic and systolic BP were considered for this study.

Maintaining all aseptic precautions, 5 mL of blood was collected following overnight fasting from the ante-capital vein of each volunteer participant by two trained laboratory technicians for biochemical analyses. The blood sample was collected using an appropriate test tube and transferred to a clean, dry serum separator tube. Then it was centrifuged at 3000 revolutions per minute (rpm) at room temperature (20 to 25°C). A pure serum sample was separated and placed in the nunc tube, where it was stored at -20 until biochemical analysis was done.

Fasting blood glucose (FBG) level, serum TC, HDL cholesterol, and TG levels were determined using Dimension EXL 200 System chemistry analyzer through enzymatic method from the serum sample using commercially available kit of auto analyzer. Serum creatinine and blood urea nitrogen (BUN) were also estimated by the enzymatic method. LDL cholesterol and VLDL cholesterol concentrations were calculated using the Friedewald formula (19). The cystatin C levels of serum were measured by a turbidimetric immunoassay test. All biochemical investigations were done on a fully automated analyzer at the National Reference Laboratory for Clinical Chemistry, Ethiopian Public Health Institute (EPHI).

### Statistical analysis

Data was entered and analyzed using SPSS version 25. Categorical variables were presented in frequency and percentages. Continuous variables including demographics, clinical and biomedical data were expressed as mean ± standard deviation. An independent sample t-test or Mann–Whitney U test was used for the comparison of variables as appropriate. An adjusted logistic regression and multiple linear regression models were employed to examine the association of predictor variables with diabetic nephropathy. All statistical tests were two-tailed, and p-values <0.05 at a 95% confidence interval (CI), were taken as statistically significant.

## Result

### The socio-demographic characteristics of the study participants

A total of 140 T2DM patients were recruited for this study (83 without diabetic nephropathy and 53 with diabetic nephropathy). The mean age of all patients was 45.2 ± 13.2 years. From all the subjects involved in the study, 74 (52.9%) were males and the rest 66 (47.1%) were females. Majority of T2DM patients 116(82.9%) were living in urban areas. In all patients, the mean ± SD of BMI was 26.3 ± 3.7 kg/m^2^. The mean ± SD of the duration of patients with T2DM was 13.3 ± 8.6 years with the maximum and minimum duration of 36 years and 1year respectively. In T2DM patients with diabetic nephropathy, we found that the TG, TC, LDL, and VLDL cholesterol levels were significantly increased, whereas HDL cholesterol levels were significantly lower (P < 0.001) in those patients. The detailed basic characteristics of the study participants are depicted in **Table 1**.

**Table 1:** Socio-demographic and clinical profiles of T2DM patients with and without nephropathy.

Variables		All T2DM patients (n=140)	T2DM patients with nephropathy (n=57)	T2DM patients without nephropathy (n=83)	p-value
Age (year), mean ± SD		45.0 ± 13.2	44.0 ± 15.12	46.2 ± 13.18	0.875
Sex	Male n (%)	74 (52.9)	32 (56.1)	42 (50.6)	0.127
	Female n (%)	66 (47.1)	25 (43.9)	41 (49.4)	0.136
Residence	Urban n (%)	116 (82.86)	48 (84.2)	68 (81.9)	0.471
	Rural n (%)	24 (17.14)	9 (15.8)	15 (18.1)	0.812
BMI, mean ± SD		26.3 ± 3.7	26.4 ± 5.98	27.5 ± 3.42	0.052
Duration of T2DM		13.3 ± 8.6	15.3 ± 7.6	12.3 ± 7.6	0.077
FBG		135 ± 14.7	175.2 ± 16.4	87.3 ± 7.1	0.071
SBP (mmHg), mean ± SD		129.2 ± 21.29	139.7 ± 19.29	128.7 ± 21.7	0.025
DBP (mmHg), mean ± SD		80.9 ± 10.24	82.9 ± 9.40	80.8 ± 11.61	0.459
TC (mg/dL) mean ± SD		197.6 ± 42.5	229.2 ± 64.8	164.7 ± 21.4	<0.001
TG (mg/dL) mean ± SD		104.8 ± 47.4.	127.8 ± 39.5	77.3 ± 56.6	<0.001
HDL (mg/dL) mean ± SD		34.7 ± 10.7	27.6 ± 8.91	41.3 ± 9.5	<0.001
LDL (mg/dL) mean ± SD		147 ± 27.1	159.3 ± 38.9	121.7 ± 24.6	<0.001
VLDL (mg/dL) mean ± SD		39 ± 11.5	51.5 ± 29.7	23.9 ± 8.5	<0.001
BUN (mg/dL) mean ± SD		29 ± 7.8	30.6 ± 8.9	26.3 ± 5.7	0.031
Scr (mg/dL) mean ± SD		0.97 ± 0.31	1.1 ± 0.4	0.8 ± 0.3	0.75
Cys-C (mg/dL mean ± SD		1.15 ± 0.6	1.3 ± 0.7	0.73 ± 0.3	<0.001

Comparisons were made using independent sample t-test, Mann-Whitney test, or Chi-square test as appropriate. Categorical variables are presented in frequency and percentage while continuous variables are presented as mean ± SD, P-value<0.05 was considered statistically significant. SD, Standard Deviation; %, percentage; BMI, Body Mass Index; SBP, Systolic Blood Pressure; DBP, Diastolic Blood Pressure; FBG, Fasting Blood Glucose; TC, Total Cholesterol; TG, Triglyceride; HDL-C, High-Density Lipoprotein Cholesterol; LDL-C, Low-Density Lipoprotein Cholesterol; VLDL, Very Low Density Cholesterol; BUN, Blood Urea Nitrogen; Cys-C, Cystatin C; Scr, Serum creatinine.

### Clinical parameters and independent predictors of diabetic nephropathy in T2DM patients

Out of 140 T2DM patients, 85 (60.7%) used oral medication, 25 (17.86%) used insulin injection, and 30 (21.4%) used both oral medication and insulin injection. After adjusting for all possible confounders using multivariable logistic regression analysis serum cystatin C (P <0.001), SBP (P = 0.02), FBG (P =0.001), TC (P <0.01), TG (P <0.001), LDL (P <0.001), VLDL (P <0.001), HDL (P<0.061) and duration of T2DM (P =0.04) were identified to be significantly associated with diabetic nephropathy. Increasing serum cystatin C levels by one unit was associated with 1.41 times (AOR: 1.41, 95% CI: 1.09, 2.77) higher odds of having diabetic nephropathy. For a unit increase in the duration of T2DM, there was a 1.32 times (AOR: 1.32, 95% CI: 1.11, 2.87) greater likelihood of having diabetic nephropathy. The odds of T2DM patients with nephropathy significantly increased per a unit rise in SBP, FBG, TC, TG, LDL, and VLDL levels (**Table 2**).

**Table 2:** Independent factors for predicting diabetic nephropathy in T2DM patients.

Variables	AOR (95% CI)	P - value
Age (in years)	0.97 (0.87-1.07)	0.381
BMI (kg/m2)	1.32 (0.44-1.89)	0.125
SBP (mmHg)	1.731 (1.01-1.072)	0.02
DBP (mmHg)	0.92 (0.95-1.022)	0.17
FBG (mg/dL)	1.30 (1.11-2.87)	0.01
TC (mg/dL)	1.5 (1.01-2.91)	<0.001
TG (mg/dL)	1.41 (1.21-2.66)	<0.001
HDL (mg/dL)	0.67 (1.23-2.57)	0.061
LDL (mg/dL)	1.21 (1.07-2.47)	<0.001
VLDL (mg/dL)	1.3 (1.11-2.87)	<0.001
BUN (mg/dL)	0.96 (0.71-1.95)	0.131
Scr (mg/dL)	0.86 (0.91-2.95)	0.07
Cys-C C (mg/dL)	1.41 (1.09-2.77)	<0.001
Diabetes duration (in years)	1.32 (1.11-2.87)	0.04

Mean + SD is significant at the p< 0.05; AOR, adjusted odds ratio; CI, confidence interval; BMI, Body Mass Index; SBP, Systolic Blood Pressure; DBP, Diastolic Blood Pressure; FB, Fasting Blood Glucose; TC, Total Cholesterol; TG, Triglyceride; HDL-C, High-Density Lipoprotein Cholesterol; LDL-C, Low-Density Lipoprotein Cholesterol; VLDL, Very Low Density Cholesterol; BUN, Blood Urea Nitrogen; Cys-C, Cystatin C; Scr, Serum creatinine.

### Comparison of clinical and biomedical parameters between males and females

An independent sample t-test showed that the mean values of TC (P = 0.021), TG (P = 0.011), and HDL cholesterol levels (P = 0.01) were significantly higher in females as compared to male type-2 diabetic patients, but significantly lower in serum creatinine levels (P = 0.03) as compared to males (**Table 3**).

**Table 3:** Comparison of clinical and biomedical parameters between males and females. .

Variables	Sex of participants		P - value
	Male (n=74)	Female (n=66)	
Age (in years)	53.7 ± 8.1	48.9 ± 5.8	0.27
BMI (kg/m^2^)	25.78 ± 5.44	23.6 ± 7.3	0.15
DBP (mmHg)	85.7 ± 10.74	82.9 ± 9.94	0.21
SBP (mmHg)	128.3 ± 20.32	125.8 ± 18.72	0.49
FBG (mg/dL)	89.9 ± 6.1	129.2 ± 16.2	0.07
TC (mg/dL)	182.7 ± 20.4	227.2 ± 63.8	0.021
TG (mg/dL)	121.8 ± 38.5	168.3 ± 76.6	0.011
HDL (mg/dL)	32.3 ± 6.5	42.6 ± 8.9	0.01
LDL (mg/dL)	138.7 ± 24.6	158.3 ± 48.9	0.501
VLDL (mg/dL)	24.9 ± 8.5	50.5 ± 28.7	0.101
BUN (mg/dL)	26.3 ± 6.7	28.6 ± 8.7	0.24
Scr (mg/dL)	1.2 ± 0.3	0.8 ± 0.4	0.03
Cys-C (mg/dL)	0.73 ± 0.2	1.3 ± 0.4	0.081

Comparisons were made using independent sample t-test, P-value<0.05 was considered statistically significant. BMI, Body Mass index; SBP, Systolic Blood Pressure; DBP, Diastolic Blood Pressure; FB, Fasting Blood Glucose; TC, Total Cholesterol; TG, Triglyceride; HDL-C, High-Density Lipoprotein Cholesterol; LDL-C, Low-Density Lipoprotein Cholesterol; VLDL, Very Low Density Cholesterol; BUN, Blood Urea Nitrogen; Cys-C, Cystatin C; Scr, Serum creatinine.

### Multiple linear regression analysis of serum cystatin C with clinical and biomedical parameters in type 2 diabetic patients

In the multiple linear regression analyses, the SBP, FPG, TC, TG, HDL, LDL, and VLDL of the T2DM participants were significantly associated with serum cystatin C levels (**Table 4**).

**Table 4:** Multiple linear regression analysis of serum cystatin C with clinical and biomedical parameters in type 2 diabetic patients.

Variables	Serum cystatin C	
	β (95% CI)	P-value
Sex of participant	-0.061	0.481
Age of participant	0.009	0.215
Duration of DM	0.066	0.303
BMI (kg/m^2^)	0.011	0.613
SBP (mmHg)	0.12	0.021
DBP (mmHg)	0.07	0.571
FPG (mg/dL)	0.54	<0.001
TC (mg/dL)	0.75	<0.001
TG (mg/dL)	0.87	<0.001
HDL (mg/dL)	-0.25	0.023
LDL (mg/dL)	0.46	0.016
VLDL (mg/dL)	0.43	0.003
BUN (mg/dL)	0.06	0.324
Scr (mg/dL)	0.032	0.451

Analysis was made using multiple linear regression analysis, P-value<0.05 was considered statistically significant. BMI, Body Mass index; SBP, Systolic Blood Pressure; DBP, Diastolic Blood Pressure; FBG, Fasting Blood Glucose; TC, Total Cholesterol; TG, Triglyceride; HDL-C, High-Density Lipoprotein Cholesterol; LDL-C, Low-Density Lipoprotein Cholesterol; VLDL, Very Low-Density Cholesterol; BUN, Blood Urea Nitrogen; Cys-C, Cystatin C; Scr, Serum creatinine.

## Discussion

This study aimed to evaluate serum cystatin C and lipid profiles for assessing the potential predictor markers of diabetic nephropathy in patients with T2DM. High serum creatinine and low cytostatin C levels were found in males, whereas low creatinine and high cystatin C levels were found in females. It is well known that the creatinine level is highly affected by muscle mass, which means that males are more muscular than females. But the cystatin C difference needs further study as it is a promising marker currently under investigation.

The study found significantly higher serum cystatin C levels and SBP and lipid profile abnormalities in type 2 diabetics with nephropathy compared to those without nephropathy. These findings are similar to other previous studies (18, 20). In contrast to these findings, the study conducted by Jing Wei et al. revealed that the serum parameters, including HDL and LDL, were not significantly different between T2DM patients with nephropathy and those without diabetic nephropathy (21). However, in the case of serum cystatin C levels, TC, and TG, this study agreed with the findings of the present study, and unlike our finding, it found a significant difference in serum creatinine in type 2 diabetics with nephropathy compared to those without nephropathy.

There is growing evidence that abnormalities in lipid metabolism contribute to renal disease progression (6). In T2DM patients with diabetic nephropathy, we found that HDL cholesterol levels were significantly low in T2DM patient with nephropathy and other parameters of lipoproteins were significantly higher. Numerous experiments studies explained the mechanism as the hyperlipidemia can cause glomerulosclerosis and tubulointerstitial sclerosis by stimulating resident renal cells to produce fibrotic cytokines and chemokines, leading to infiltration of monocytes or macrophages into glomerular tissue to promote extracellular matrix deposition and have shown that lipid accumulation, lipid toxicity and lipid metabolism disorders are related to diabetic kidney damage (6, 22).

Several studies found that the levels of serum cystatin C rose earlier than creatinine (22, 23). In this study we found that the cystatin C level of the type 2 DM is increased significantly and associated with lipoproteins in T2DM patients diagnosed nephropathy. This showed that the serum cystatin C level is related to subclinical tubular impairment and can be an earlier marker of renal disease in diabetic patients before the onset of other renal markers. This is in agreement with some other earlier studies (23, 24).

In the multivariate regression analysis, we found that the serum cystatin C level is significantly associated with FBG, TC, TG, HDL, LDL, and VLDL (P < 0.001). However, it is insignificantly associated with serum creatinine, duration of DM, BMI, BUN, and the age of the participant. This was in line with study conducted by Kachhawa et al. (18).

This study also revealed the mean values of TC, and TG were significantly higher (p < 0.05) in females as compared to male type-2 diabetic patients, but low in HDL and serum creatinine levels as compared to males. Another study conducted by Radzeviciene et al., support these results on type one diabetic patients (19, 25).

## Conclusion

We found that serum cystatin C is not significantly affected by sex, unlike serum creatinine, and a significant difference was found between T2DM patients with nephropathy and those without nephropathy. The present study also found significant lipoprotein abnormalities in T2DM patients with diabetic nephropathy when compared with those without diabetic nephropathy, and these lipoprotein abnormalities were significantly associated with serum cystatin C levels.

## Data availability statement

The original contributions presented in the study are included in the article/supplementary material. Further inquiries can be directed to the corresponding author.

## Ethics statement

The studies involving human participants were reviewed and approved by Addis Ababa University Department of Ethics and Research Committee (DRERC). The patients/participants provided their written informed consent to participate in this study.

## Author contributions

All authors made a significant contribution to the work reported, whether that is in the conception, study design, execution, data acquisition, analysis, and interpretation, or all these areas; took part in drafting, revising, or critically reviewing for important intellectual content. All authors have agreed on the journal to which the article has been submitted; and agreed to be accountable for all aspects of the work. All authors contributed to the article and approved the submitted version.
